# Aortoesophageal fistula 19 years after radiotherapy for oesophageal cancer: a case report

**DOI:** 10.1093/ehjcr/ytae612

**Published:** 2024-11-16

**Authors:** Satoshi Sakakibara, Akira Marumoto

**Affiliations:** Department of Cardiovascular Surgery, Higashiosaka City Medical Center, 3-4-5, Nishiiwata, Higashiosaka, Osaka 578-8588, Japan; Department of Cardiovascular Surgery, Higashiosaka City Medical Center, 3-4-5, Nishiiwata, Higashiosaka, Osaka 578-8588, Japan

**Keywords:** Aortoesophageal fistula, Oesophageal cancer, Radiotherapy, Case report

## Abstract

**Background:**

Aortoesophageal fistula (AEF) is a life-threatening disease that causes massive bleeding, sepsis, and ultimately death. Therefore, emergency treatments are required. Recently, cases of AEF treated with thoracic endovascular aortic repair (TEVAR) have been reported.

**Case summary:**

An 84-year-old man was referred to a local hospital with massive haematemesis and transient loss of consciousness. The patient’s medical history included radiotherapy for oesophageal cancer 19 years before presentation. Gastrofiberscopy revealed an ulcer in the thoracic oesophagus and no recurrence of oesophageal cancer. Computed tomography (CT) showed that the same area was adjacent to the thoracic aorta and that there was no thoracic aortic aneurysm. The patient was then transferred to our institution for surgical treatment. We diagnosed the patient with an AEF caused by radiotherapy of the oesophagus. Therefore, TEVAR was performed. Due to the patient’s advanced age, condition, and the possibility of strong adhesions, there was a high risk that they would be unable to tolerate oesophagectomy to prevent stent graft infection. Therefore, antibiotic therapy was initiated. However, CT revealed a stent graft infection 24 days after TEVAR. The patient died of sepsis 27 days after the procedure.

**Discussion:**

We describe the first case of AEF as a very late complication of radiotherapy for oesophageal cancer. Surgery to repair oesophageal defects is necessary to prevent stent graft infection. However, the decision for such surgery should be made on a case-by-case basis, taking into account the patient’s condition and ability to tolerate the procedure.

**Learning points**

- Aortoesophageal fistula, as a late complication of radiotherapy for oesophageal cancer, is rare and requires surgical treatment, such as a combination of thoracic endovascular aortic repair and oesophageal defect repair.
- The decision for such surgery should be made on a case-by-case basis, considering the patient’s condition and ability to tolerate the procedure.

## Introduction

Aortoesophageal fistula (AEF) is a life-threatening disease that causes massive bleeding, sepsis, and ultimately death.^1^ Therefore, emergency treatments are required. Aortoesophageal fistula can be caused by oesophageal cancer (EC), thoracic aortic aneurysm (TAA), foreign bodies, and radiotherapy (RT), among other factors.^1^ Aortoesophageal fistula caused by RT develops relatively early, within 1 year after therapy. Open surgery for AEF is associated with high mortality and morbidity rates. Recently, the use of thoracic endovascular aortic repair (TEVAR) to treat AEF has been reported. Herein, we report a case of TEVAR for an AEF 19 years after chemoradiotherapy for EC.

## Summary figure

**Table:** 

Timeline	
19 years prior to the AEF diagnosis	Chemoradiotherapy was performed for oesophageal cancer
AEF diagnosis	AEF diagnosis by gastrofiberscopy (GF) and computed tomography (CT)
Treatment	TEVAR using CTAG and antibiotic therapy

## Case report

**Figure 1:**
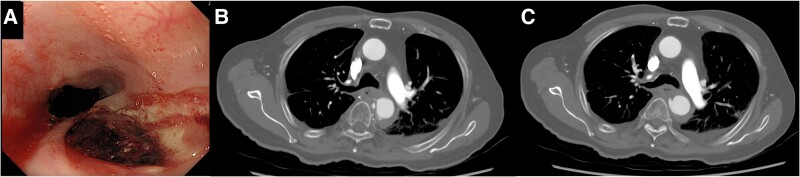
Gastrofiberscopy revealed an ulcer in the thoracic oesophagus (*A*), and computed tomography revealed that the same area was adjacent to the thoracic aorta (*B* and *C*).

An 84-year-old man with haematemesis and transient loss of consciousness was referred to a local hospital. The patient’s medical history included chemoradiotherapy with 60 Gy for EC 19 years prior, chronic pericardial effusion caused by RT, cholecystectomy for gallstones, hypertension, dyslipidaemia, and dementia. His clinical frailty scale score was 5. Gastrofiberscopy revealed an ulcer in the thoracic oesophagus, and CT revealed the same area adjoining the thoracic aorta (*Figure 1*). The patient was diagnosed with an AEF and transferred to our institution for surgical treatment. Physical examination upon admission revealed a regular pulse of 77 b.p.m., a blood pressure of 102/51 mmHg, and a body temperature of 36.2°C. Blood tests revealed a white blood cell count of 14 900 per cubic millimetre with 87.6% neutrophils, a haemoglobin level of 11.4 g/dL, a platelet count of 243 000 per microlitre, and a C-reactive protein (CRP) level of 5.24 mg/dL. Renal and liver function tests were within normal limits. Gastrofiberscopy and CT showed no recurrence of EC, TAA, foreign bodies, or abscesses. Therefore, the patient was diagnosed with AEF caused by RT for EC and planned TEVAR.

**Figure 2:**
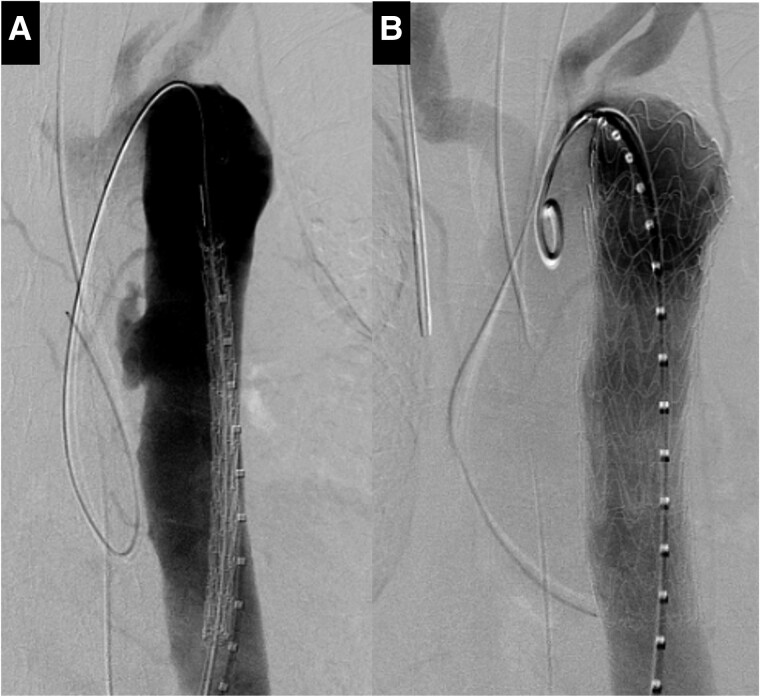
Aortography before thoracic endovascular aortic repair showed the fistula as an irregularity of the descending aorta (*A*). The final aortography showed no endoleakages (*B*).

General anaesthesia was induced. An 8-Fr sheath was inserted through the exposed left common femoral artery. After the administration of heparin, aortography revealed an irregular fistula in the descending aorta (*Figure 2A*). A stent graft (CTAG; W.L. Gore and Associates, Flagstaff, AZ, USA; diameter, 31 mm; length, 100 mm) was used to cover the fistula. Subsequently, a stent graft (CTAG; diameter: 37 mm; length: 100 mm) was placed in the distal aortic arch. Final aortography showed no endoleaks (*Figure 2B*). The total operative time was 62 min. Blood loss was low, and transfusion was not required. The patient was then extubated in the operating room.

**Figure 3:**
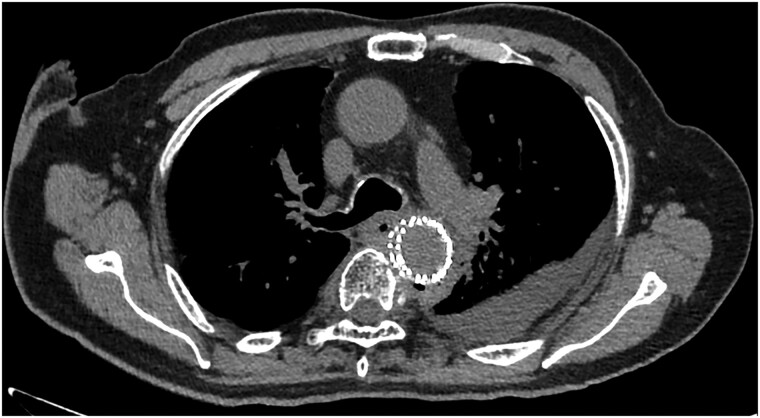
Because postoperative computed tomography revealed the air around the stent graft, the patient was diagnosed with stent graft infection 25 days after thoracic endovascular aortic repair.

Postoperatively, meropenem (1.0 g three times daily) was initiated. On postoperative day 5, drug-induced liver injury caused by meropenem was revealed, prompting a switch to tazobactam/piperacillin (4.5 g four times daily). Postoperative CT showed no endoleaks or significant findings indicating stent graft infection 7 days after TEVAR. In addition, postoperative GF did not show that the stent graft was exposed on the oesophageal side. Therefore, oral fluid was resumed 15 days after TEVAR. Blood cultures were negative, sputum cultures were positive for normal flora, and β-D glucan levels were within normal limits. Consequently, the antibiotic was changed to sulbactam/ampicillin (3.0 g three times daily) 18 days after TEVAR. On the 25th day after TEVAR, the patient developed a fever, and CT revealed air around the stent graft (*Figure 3*). As the patient was diagnosed with a stent graft infection, the antibiotic was changed to tazobactam/piperacillin. The patient died of sepsis caused by a stent graft infection 27 days after TEVAR.

## Discussion

Aortoesophageal fistula is rare, and its main aetiologies include TAA (54.2%), foreign body (19.2%), oesophageal cancer (17%), RT, and infection.^1^ In terms of RT, radiation doses higher than 35 Gy are a risk factor,^1^ resulting in AEF in 8.3% of patients with ECs treated with RT.^2^  *Table 1* shows the clinical features of patients in previous studies and those in the present case.^1–12^ Of the 16 patients, 13 were male. Their average age was 65.4 years (range 42–81 years). The timing of AEF onset was 0–10 months after RT. Aortoesophageal fistula is a life-threatening disease that causes massive bleeding, sepsis, and ultimately death.^1^ Therefore, emergency and aggressive treatments are required. The treatment of AEF requires surgery to remove infected lesions, revascularize, and prevent reinfection. In accordance with EACTS/STS guidelines for diagnosing and treating acute and chronic aortic syndromes, concomitant radical oesophagectomy with proximal cervical oesophagostomy, pyloromyotomy, and gastrostomy for nutritional support has been widely accepted and remains the most successful approach reported.^13^ However, patients with AEF are often in poor condition, and open surgery for AEF is associated with high mortality. Recently, TEVAR has been reported as a minimally invasive treatment. Thoracic endovascular aortic repair for AEF is useful for the rapid and effective control of bleeding. In addition, this treatment has advantages, such as no median sternotomy, less damage, no aortic clamp, and less cerebral ischaemic damage.^1^ For these reasons, TEVAR for AEF contributes to improved postoperative survival, with a technical success rate of 87.3% and a 30-day mortality rate of 19.7%.^14^ However, as long as the oesophageal defect remains, the stent graft remains exposed to the infection site, often leading to death from stent graft infection (15.2%), recurrent fistula (13.8%), mediastinitis, or sepsis.^14^ After TEVAR, repair of the oesophageal defect is necessary to prevent these complications. There is no consensus on the optimal timing for oesophageal defect. In terms of these methods, primary repair with a direct suture or patch of oesophageal erosion is possible only in cases of small oesophageal defects without gross contamination of the mediastinum.^14^ Otherwise, oesophageal resection should be considered as the treatment of choice in most patients with AEF. As a nonsurgical method, antibiotics administration for over 6 weeks^13^ and oesophageal stent implantation to prevent contact of the stent graft with the septic environment may have potential benefits.^4^ However, oesophageal stent is associated with a risk of chest pain, recurrent fistulas, and mediastinitis.^15^ Therefore, oesophageal stent implantation should be performed in selected patients.

**Table 1:** Reported cases of aortoesophageal fistula caused by radiotherapy for oesophageal cancer

Authors	Year	Age	Sex	Days to AEF	RT dose	Operation	Outcome	Survival period	Cause of death
Negoto *et al*.^3^	2023	71	Male	13 days	60 Gy	TEVAR, gastrostomy	Death	103 days	Cancer
Owczarek *et al*.^4^	2023	70	Male	3 days	64 Gy	TEVAR	Death	3 months	Cancer
Zhong *et al*.^1^	2022	66	Male	10 months	60 Gy	TEVAR	Death	2 months	Cancer
Hayashi *et al*.^5^	2022	49	Female	7 months	36 Gy	TEVAR, Oesophagectomy, aortic replacement, gastrointestinal reconstruction	Alive	174 days	–
Guerrero *et al*.^6^	2020	69	Male	Unknown	Unknown	TEVAR	Death	3 h	Haematemesis
Iwabu *et al*.^7^	2020	69	Male	16 days	60 Gy	TEVAR	Death	7 months	Cancer
Ikegaya *et al*.^8^	2019	67	Male	2 months	59.4 Gy	TEVAR	Death	115 days	Cancer
Sasaki *et al*.^9^	2018	67	Male	Unknown	59.4 Gy	TEVAR	Death	4 months	Cancer
Fujiwawa *et al*.^10^	2017	42	Male	Unknown	Unknown	TEVAR	Death	56 days	Cancer
Ishikawa *et al*.^11^	2013	75	Female	1 month	60 Gy	TEVAR	Death	4 months	Sepsis
Ishikawa *et al*.^11^	2013	81	Male	3 months	60 Gy	TEVAR, oesophageal stent	Alive	1 year	–
Taniguchi *et al*.^2^	2011	59	Male	88 days	60 Gy	Conservative	Death	0 days	Haemorrhage
Taniguchi *et al*.^2^	2011	59	Male	82 days	60 Gy	Conservative	Death	5 days	Haemorrhage
Taniguchi *et al*.^2^	2011	71	Female	83 days	60 Gy	Conservative	Death	0 days	Haemorrhage
Taniguchi *et al*.^2^	2011	73	Male	49 days	52 Gy	Conservative	Death	1 days	Haemorrhage
Kato *et al*.^12^	2000	59	Male	During RT	58 Gy	TEVAR, enterostomy	Death	135 days	Pneumonia
Our case	–	84	Male	19 years	60 Gy	TEVAR	Death	27 days	Sepsis

In our case, chemoradiotherapy was performed for EC 19 years ago, and the patient was cured. At the time of AEF, GF and CT revealed no significant findings indicating TAA, EC recurrence, foreign bodies, or infection. Therefore, we considered AEF as a late complication of RT for EC. Thoracic endovascular aortic repair was effective in controlling bleeding. Surgery for oesophageal defects is considered to prevent stent graft infection. However, due to the patient’s advanced age, condition, and potential strong adhesions after RT, we considered that the patient could not tolerate surgery for an oesophageal defect. Therefore, broad-spectrum antibiotics were initiated empirically, and strict food fasting was followed. Since the cause of AEF was tissue fragility resulting from RT for EC, we considered that oesophageal stent implantation could increase the risk of oesophageal perforation, including recurrence of AEF, and therefore, it was not performed. Additionally, the patient developed severe postoperative delirium, making the insertion of a jejunostomy tube to support oesophagus healing difficult. Postoperative CT performed 7 days after TEVAR showed no endoleaks or stent graft infection, and GF 14 days after TEVAR confirmed closure of the oesophageal defect. The patient resumed oral fluid 15 days after TEVAR. Postoperative antibiotics were initiated and adjusted based on blood and sputum culture results, as well as β-D glucan levels. However, the patient developed a fever, and the CRP level increased again. The second postoperative CT scan, which was performed 25 days after TEVAR, revealed a stent graft infection, and the patient died of sepsis 2 days later.

This report describes a case of AEF caused as a late complication of RT for EC. Thoracic endovascular aortic repair effectively controlled the bleeding. Surgery to repair oesophageal defects is necessary to prevent stent graft infection. However, the decision for such surgery should be made on a case-by-case basis, taking into account the patient’s condition and ability to tolerate the procedure.

## Lead author biography


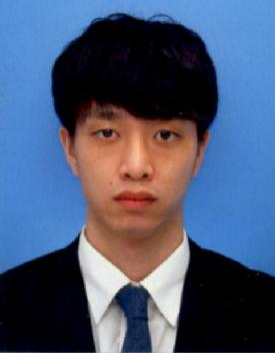


The lead author graduated from Kyorin University in 2015 and currently works as a cardiovascular surgeon at Higashiosaka City Medical Center, Japan. His areas of interest include aortic diseases.

##  

**Consent:** The authors confirm that written consent for submission and publication of this case report, including the images and associated text, is in agreement with the COPE guidelines.

**Funding:** None declared.

## Data Availability

All data underlying the results are available as part of the article, and no additional source data are required.
